# Age- and Gender-Related Differences in LDL-Cholesterol Management in Outpatients with Type 2 Diabetes Mellitus

**DOI:** 10.1155/2015/957105

**Published:** 2015-03-19

**Authors:** Giuseppina Russo, Basilio Pintaudi, Carlo Giorda, Giuseppe Lucisano, Antonio Nicolucci, Maria Rosaria Cristofaro, Concetta Suraci, Maria Franca Mulas, Angela Napoli, Maria Chiara Rossi, Valeria Manicardi

**Affiliations:** ^1^Department of Internal Medicine, University of Messina, 98125 Messina, Italy; ^2^Department of Clinical Pharmacology and Epidemiology, Fondazione Mario Negri Sud, Via Nazionale, 66030 S. Maria Imbaro, Italy; ^3^Diabetes and Metabolism Unit, ASL TO5, 10023 Chieri, Italy; ^4^Diabetes and Endocrinology Unit, Cardarelli Hospital, 86100 Campobasso, Italy; ^5^Diabetes and Metabolism Unit, Sandro Pertini Hospital, 00157 Rome, Italy; ^6^Diabetes and Metabolic Diseases Unit, San Martino Hospital, 09170 Oristano, Italy; ^7^Department of Clinical and Molecular Medicine, Faculty of Medicine and Psychology, S. Andrea Hospital, Sapienza University, 00189 Rome, Italy; ^8^Diabetes Unit, Montecchio Hospital, 42027 Montecchio Emilia, Italy

## Abstract

*Background*. Dyslipidemia contribute to the excess of coronary heart disease (CHD) risk observed in women with type 2 diabetes (T2DM). Low density lipoprotein-cholesterol (LDL-C) is the major target for CHD prevention, and T2DM women seem to reach LDL-C targets less frequently than men. *Aim*. To explore age- and gender-related differences in LDL-C management in a large sample of outpatients with T2DM. *Results*. Overall, 415.294 patients (45.3% women) from 236 diabetes centers in Italy were included. Women were older and more obese, with longer diabetes duration, higher total-cholesterol, LDL-C, and HDL-C serum levels compared to men (*P* < 0.0001). Lipid profile was monitored in ~75% of subjects, women being monitored less frequently than men, irrespective of age. More women did not reach the LDL-C target as compared to men, particularly in the subgroup treated with lipid-lowering medications. The between-genders gap in reaching LDL-C targets increased with age and diabetes duration, favouring men in all groups. *Conclusions*. LDL-C management is worst in women with T2DM, who are monitored and reach targets less frequently than T2DM men. Similarly to men, they do not receive medications despite high LDL-C. These gender discrepancies increase with age and diabetes duration, exposing older women to higher CHD risk.

## 1. Introduction

Type 2 diabetes (T2DM) is a powerful cardiovascular disease (CVD) risk factor in both men and women. Although the overall CVD risk is higher in T2DM men, the relative risk of coronary heart disease (CHD) is higher in T2DM women when compared to nondiabetic ones, with the loss of the typical oestrogen protection in the premenopausal state [1–3].

Although the mechanism underlying this excessive CHD risk in women with type 2 diabetes (T2DM) has not been fully elucidated yet, several hypotheses suggest that diabetes* per se* may be a stronger CHD risk factor in the female gender, determining a more unfavourable CHD risk profile [4, 5]. This could lead to more complex risk factors and/or disease management in women with T2DM as compared to men.

Chronic hyperglycemia may certainly play a role, but it is not the only responsible for the high CHD burden in subjects with T2DM. Thus, cardiovascular disease (CVD) is a multifactorial condition and major risk factors (i.e., obesity, hypertension, and dyslipidemia) have been all demonstrated to contribute to its occurrence [6].

Among these risk factors, low density lipoprotein-cholesterol (LDL-C) is a major target for CVD prevention, also in subjects with T2DM. Thus, the UK Prospective Diabetes Study (UKPDS) demonstrated that LDL-C is the stronger CVD risk factor in subjects with T2DM, even when compared to glycated haemoglobin (HbA1c) and hypertension [7]. Furthermore, several intervention trials with statins have demonstrated the beneficial effect of lowering LDL-C in both primary and secondary CVD prevention, especially in subjects with T2DM [8, 9].

Circulating lipid fractions, including LDL-C, are strongly affected by individual characteristics, including age and gender, but these differences are not always taken into account when managing lipid disorders. In this regard, in a large cohort of Italian T2DM outpatients, we have recently reported that T2DM women were 42% more likely to have LDL-C above the recommended targets, in spite of lipid-lowering treatment and in the context of an overall lower quality of care [10]. Thus, LDL-C levels usually increase with age in men, whereas high density lipoprotein-cholesterol (HDL-C) concentrations tend to decrease with ageing in men, but not in women [11], leading to a ~10 mg/dL between-gender difference in HDL-C levels [12]. Furthermore, gender-related differences have been reported in the pathophysiology, diagnosis, and treatment of lipid disorders.

Since LDL-C is the major goal of CHD prevention in subjects with T2DM, here we further analyzed data on LDL-C management in the large cohort of the AMD Annals initiative [10], in order to assess potential differences according to gender, age, and diabetes duration.

## 2. Materials and Methods

The population characteristics and study design have been described in detail elsewhere [10]. Briefly, information recorded on electronic medical records between January 1, 2009, and December 31, 2009, of a large sample of patients with a diagnosis of T2DM attending 236 diabetes clinics in Italy was considered. Approximately, one-third of all the Italian diabetes clinics, uniformly distributed throughout the country, were involved in the present study.

The used database derives from the Italian Association of Clinical Diabetologists (Associazione Medici Diabetologi (AMD)) initiative which started in 2006 with the aim of monitoring quality of diabetes care [13, 14]. Its objective was to identify a set of indicators to be used in the context of continuous quality improvement. All participating clinics used an electronic clinical record system for the everyday management of outpatients, and software was specifically developed to extract information from all these clinical databases (AMD data). Data from all participating clinics were collected anonymously and were centrally analysed [13, 14].

The core data set included measures and monitoring of HbA1c, blood pressure, BMI, total-cholesterol, LDL-C or HDL-C, and triglycerides. The use of specific classes of drugs (glucose-lowering, lipid-lowering, and antihypertensive agents), based on ATC codes, was also evaluated.

Current smokers were identified in the electronic clinical record based on a specific Yes/No field. In the case of multiple evaluations of the considered parameters during the year, the most recent ones were taken into consideration for the analysis.

### 2.1. Statistical Analysis

Clinical characteristics are expressed as mean and standard deviation for continuous variables and frequencies and percentages for categorical ones. The entire sample of studied subjects was divided into two groups according to gender. Further analyses were performed evaluating between-group gender-related differences after stratifying for age, diabetes duration, or both. Some between-gender disparities were also expressed as absolute differences. Even if trivial differences would have reached statistical significance, due to the large sample size, between-group nonparametric statistical tests were applied. All statistical analyses were performed using SAS version 9.2 (SAS Institute Inc.).

## 3. Results 

Overall, a total of 415.294 subjects with T2DM followed by 236 diabetes outpatient centers were evaluated. Clinical characteristics of subjects with T2DM included in the analysis according to gender are shown in Table 1. Overall, 45.3% of participants were women. Women with T2DM were older, smoked less, and had a longer diabetes duration and higher BMI, compared with T2DM men (*P* < 0.0001 for all comparisons), whereas they had similar systolic and diastolic blood pressure levels. Women also showed higher HbA1c levels compared to men (*P* < 0.001), except when considering younger subjects (age class <55 years) and a shorter diabetes duration (<2 years) (data not shown). Analysis of lipid profile revealed that women had significantly higher mean total-cholesterol (T-C), LDL-C, and HDL-C serum levels compared to men (*P* < 0.0001 for all comparisons). Serum triglycerides levels were not statistically different between groups. Conversely, LDL/HDL ratio was slightly, but significantly, higher in T2DM men than in women (*P* < 0.0001).

Despite these differences in lipid profile, the use of lipid-lowering medications was overall low and equally distributed in both genders (41.2% of study population). Table 1 shows that, among lipid-lowering drugs, the vast majority of patients (38% of the women and 37% of the men, *P* < 0.0001) were treated with statins; the use of omega 3 fatty acids and fibrates was lower in women than in men (*P* < 0.0001), and very few patients were treated with ezetimibe or bile acids sequestrants in both genders.

**Figure 1:**
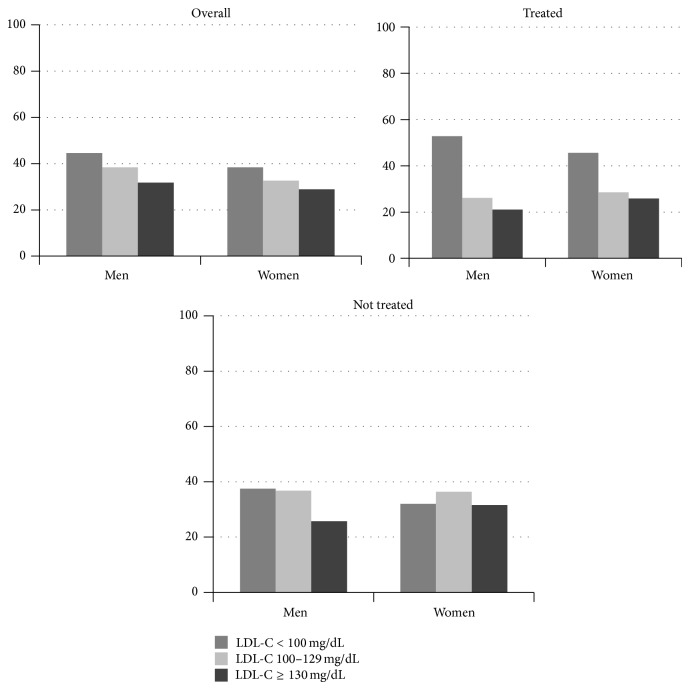
LDL-C classes according to gender and lipid-lowering treatment.

As shown in Figure 1, when stratifying study population according to LDL-C targets, a lower percentage of women had LDL-C levels within the recommended values <100 mg/dL as compared to men, whereas the percentage of subjects with LDL-C ≥130 mg/dL was higher in women than in men.

These disparities were also evident when considering patients not taking lipid-lowering medications (subjects with LDL-C <100 mg/dL: 32.0% women versus 37.5% men, respectively; subjects with LDL-C ≥130 mg/dL: 31.6% women versus 25.7% men, respectively, *P* < 0.0001). Notably, these gender disparities were even more striking in the subgroup of subjects treated with lipid-lowering medications, since the between-genders gap in reaching the LDL-C target of <100 mg/dL was of 7.2% (45.6% versus 52.8% for women and men, resp.), despite the fact that a similar percentage of men and women were treated with lipid-lowering drugs, and a high proportion of women were treated with statins.

### 3.1. LDL-C Management in T2DM Outpatients according to Gender and Age

Since both age and gender may profoundly affect lipid values, we evaluated several indicators of LDL-C management according to these parameters, stratifying our population in 4 age groups, each with a mean age that was comparable in T2DM men and women (Table 2).

Overall, lipid profile was monitored in about 75% of study subjects, more commonly in the middle age groups (55–75 yrs), whereas those older than 75 years showed the least frequency of monitoring. In each subgroup, women were always monitored less frequently than men, irrespective of age. Furthermore, women did not reach the LDL-C target of <100 mg/dL as men, and this between-genders gap increased with ageing, going from 3.1% in younger subjects to 8.3% in those >75 years old. Accordingly, subjects with higher LDL-C levels (≥130 mg/dL) were more frequent among female participants in all age groups.

### 3.2. LDL-C Management in T2DM Outpatients according to Gender and Diabetes Duration

Data on LDL-C management were also analyzed according to diabetes duration, which was slightly longer in women than in men (Table 3). Women were 2-3 years older than men in each diabetes duration group. Also, with this stratification, women were less frequently monitored for lipid profile and reached targets less frequently than men. This between-genders gap in favour of men increased along with diabetes duration, going from 5.5% for individuals with a recent diagnosis of diabetes to 7.3% in those with a diabetes duration >10 yrs. Accordingly, when considering the percentages of subjects with a LDL-C ≥130 mg/dL, women showed higher percentages compared with men, across all diabetes duration groups.

### 3.3. LDL-C Management in T2DM Outpatients according to Gender, Age, and Diabetes Duration

After adjusting for age and diabetes duration (Table 4), LDL-C management was worst in women with T2DM, who were less frequently monitored for lipid profile and less frequently reached LDL-C targets as compared with men. Furthermore, subjects with T2DM of both genders did not receive lipid-lowering medications despite high LDL-C levels in 57.5% of cases.

## 4. Discussion

Diabetes is a powerful CVD risk factor. Although the absolute risk is higher for men (2.0%) than for women (1.2%), the relative risk of CHD events from having T2DM is higher in women. A meta-analysis of 37 prospective cohort studies showed that the relative risk of fatal coronary heart disease associated with diabetes was higher in women than in men when compared to their nondiabetic counterparts [2]. Thus, the rate of fatal coronary heart disease was substantially higher in people with diabetes than in those without (5.4% versus 1.6%), but this difference, which was apparent in both sexes, was more evident among women (7.7 versus 1.2% in those with and without diabetes), whereas the corresponding rates in men were 4.5% and 2.0% [2]. Furthermore, Kalyani and colleagues studied three large cohorts of subjects and found that the risk of incident and fatal CHD in young and middle-aged women with diabetes increased by four- to fivefold as compared to those without diabetes, but these differences were not shown in men [15].

In the recent years, the necessity to consider separately men and women in the process of care was increasingly evident [16]. Gender medicine describes differences in the way of interpreting risk factors, clinical manifestations, and therapeutic approaches in men and women; a better knowledge of these aspects could address prevention and therapeutic strategies, as endorsed by the American Heart Association that recently established specific indications for women [17].

While awaiting an elucidation of the pathophysiological bases of these gender-related differences in CVD, the systematic correction of major modifiable risk factors, that is, a composite control of HbA1c, blood pressure, and LDL-C levels, represents the only strategy to lower CVD risk in patients with T2DM of either sexes.

In a large sample of outpatients with T2DM regularly attending over 200 diabetes units nationwide, we have recently shown that women were more likely to have out-of-target LDL-C levels and an overall poorer quality of diabetes care, as compared to men [10]. Thus, T2DM women were 14% more likely than men to have HbA1c ≥9.0%, 42% more likely to have LDL-C ≥130 mg/dL, and 50% more likely to have BMI ≥30 kg/m^2^; they were also less likely to be monitored for foot and eye complications.

Since LDL-C is the major target for CVD primary and secondary prevention, also in subjects with T2DM [12], the present study was aimed at investigating how this risk factor is managed in our routine diabetes outpatient activity and whether age, gender, and diabetes duration may influence it.

Although our current paper and that by Rossi et al. [10] consider the same population of subjects with T2DM, they differ in aims, analysis, and outcomes. Rossi and colleagues [10] aimed to evaluate sex differences in pharmacological and nonpharmacological treatment of diabetes and to investigate the role of biological and cultural factors in determining different outcomes for men and women. They performed multilevel regression analyses, taking into account clustering effect, and explored intercenter variability [10]. Conversely, the aim of our present analysis was to focus on potential age- and gender-related differences in lipid management in the routinary care of T2DM outpatients. Therefore, in spite of the simpler statistical analyses used, our study represents a deeper investigation on a particular point the previous paper did not specifically focus on. Our results showed that T2DM women are more likely than men to have LDL-C levels above treatment goals, thus confirming in a larger cohort the results of other studies on this field [2, 18, 19]. This finding was very consistent, since it was shown across subgroups with varying age and duration of T2DM, but also when considering subjects taking or not taking lipid-lowering medications, separately.

The higher LDL-C values in T2DM women not taking lipid-lowering medications may reflect higher basal lipid values in this group, that is, their natural tendency to a worse lipid profile. It is well recognized that lipid profile may vary in women according to age and peculiar biological phases, such as pregnancy and menopause, which are both associated with a more atherogenic lipid profile, with higher total-cholesterol (T-C), LDL-C, and triglycerides concentrations [20–25].

Overall, our data indicate that the gender gap in reaching LDL-C targets increased with ageing and diabetes duration, with older T2DM women (≥75 yrs old) and those with a longer diabetes duration (>10 yrs) showing the greatest (~10%) difference as compared to T2DM men.

Nevertheless, the most striking finding of our study is that T2DM women were not able to reach the recommended LDL-C targets as men, in spite of a similar rate in the use of medications and a slightly higher use of statins. These differences were observed in each age group and in spite of diabetes duration. Several reports have shown gender-related differences in the levels of major CVD risk factors in T2DM cohorts [2, 18, 19] and our data are in agreement with most of these studies, indicating a worse CVD risk profile in T2DM women, despite a similar rate in the use of specific medications. Furthermore, we extended these observations by showing that older women with a longer diabetes duration are those at higher risk of being inappropriately treated. This finding is particularly relevant in the light of the extremely high CHD risk of older T2DM women [26].

Although the cross-sectional nature of our evaluation does not allow us to investigate the causal relationships underlying these gender-related differences in LDL-C management, several possible explanations could be taken into account. Thus, it is likely that, despite similar prescriptions, women are less adherent to treatments [27] or that a treatment bias in favour of men exists. In the latter case, men could receive a better quality of care, with better therapies and, generally, a more complete care [28]. Despite these considerations, it is important here to underline that the burden of CHD is still very high in T2DM men, who are those exposed to the overall higher risk of developing fatal and nonfatal CHD events.

We can also hypothesize that LDL-C levels are different because of a possible different titration of lipid-lowering drugs.

This latter hypothesis would explain some of our results, since women with T2DM in our study were less frequently monitored than men, irrespective of age and diabetes duration. In general, women could also have more social barriers and a lower understanding of the importance of their CVD risk, compared with men. This low perception could be worsened by the presence of stress and lack of time for self-care due to childcare, eldercare, and increasing work activities [29]. All those factors could lead to a lower adherence to treatment in women, as documented in a recent European experience, in which a medication assessment tool was employed to test adherence of patients to evidence-based clinical prescribing recommendations [30]. The other intriguing hypothesis explaining the higher risk of having worse lipid profiles in women compared with men could be a gender-specific lipid-lowering resistance. Sex-specific differences in the pharmacokinetics and pharmacodynamics of drugs are still unclear [27]. Moreover, in the last years, several genes-drugs interactions influencing statins responses were reported [18]. Notably, T2DM men and women may be strikingly different also for lipoprotein subclasses composition and function not limited to LDL-C [31], and, in our study, LDL/HDL ratio was slightly, but significantly, higher in T2DM men than in women. This measure integrates information on both LDL-C and HDL-C, and higher values indicate a higher prevalence of circulating atherogenic particles.

In this regard, another interesting point is the overall low use of nonstatin lipid-lowering medications such as ezetimibe, bile acids sequestrants, omega 3 fatty acids, or fibrates, in the whole cohort and especially in women, who have been reported to be particularly susceptible to the effects of atherogenic dyslipidemia, that is, low HDL-C and high triglycerides levels. Thus, several reports have indicated that CVD risk in T2DM women could depend upon non-LDL-C factors, including atherogenic dyslipidemia or more subtle alterations of lipid profile [31–34]. The modest use of these drugs in women may contribute to the failure to achieve the non-LDL-C lipid targets and to their overall high CVD risk.

## 5. Conclusions 

Our study has several strengths, that is, the large sample size of individuals with T2DM evaluated and the data source utilized. Accordingly, although not comprising all Italian T2DM subjects, our study may be considered a good representation of the quality of diabetes care in Italy. The lack of information on drug doses, sociodemographic and socioeconomic characteristics, and diabetes complications and the impossibility of generalizing our findings to patients cared for by general practitioners may represent limitations of our study.

In conclusion, our data confirm a gender disparity in the routine management of LDL-C levels in T2DM outpatients. Health care professionals should advise women with T2DM about their potential CVD risk, and should not give priority only on treating hyperglycemia and diabetes-related symptoms. Clinicians should also take into account specific gender-related conditions, particularly those capable of influencing CVD risk, with the aim of personalizing their therapeutic actions.

## Figures and Tables

**Table 1:** Clinical characteristics of T2DM outpatients according to gender.

	Female	Male	*P*
*n* (%)	188,125 (45.3)	227,169 (54.7)	
Age (yrs)	68.4 ± 11.4	65.7 ± 11.1	<0.0001
Smokers (%)	11.8	21.5	<0.0001
Diabetes duration (yrs)	11.1 ± 9.8	10.0 ± 9.1	<0.0001
BMI (kg/m^2^)	30.2 ± 5.9	29.2 ± 4.6	<0.0001
Systolic blood pressure (mmHg)	139.9 ± 19.4	138.6 ± 18.7	<0.0001
Diastolic blood pressure (mmHg)	78.9 ± 9.7	79.3 ± 9.8	<0.0001
HbA1c (mmol/mol)	58 ± 16.4	57 ± 16.4	<0.0001
Total-cholesterol (mg/dL)	194.4 ± 40.9	182.3 ± 40.8	<0.0001
LDL-cholesterol (mg/dL)	112.5 ± 34.8	106.6 ± 34.4	<0.0001
HDL-cholesterol (mg/dL)	53.3 ± 14.0	46.3 ± 12.6	<0.0001
LDL/HDL ratio	2.2 ± 0.9	2.4 ± 1.0	<0.0001
Triglycerides (mg/dL)	143.4 ± 88.3	151.7 ± 121.6	0.40
Patients treated with lipid-lowering medications (%)	41.2	41.2	0.74
Statins use (%)	38.2	37.2	<0.0001
Omega 3 use (%)	1.9	2.4	<0.0001
Fibrates use (%)	3.9	6.5	<0.0001
Ezetimibe use (%)	0.1	0.0	0.22
Bile acids sequestrants use (%)	0.1	0.0	0.12

Data are *n*, %, and means ± standard deviation.

**Table 2:** Management of LDL-C values in T2DM outpatients according to gender and age.

	<55 years			55–65 years			65–75 years			>75 years		
	F	M	*P*	F	M	*P*	F	M	*P*	F	M	*P*
*n*	22069	37422		42914	62970		65535	79415		57230	47210	

Age (yrs)	47.1 ± 7.3	47.7 ± 6.4	<0.0001	60.6 ± 2.7	60.4 ± 2.8	<0.0001	70.1 ± 2.8	69.9 ± 2.8	<0.0001	80.6 ± 4.1	79.8 ± 3.7	<0.0001

Diabetes duration (yrs)	6.6 ± 7.2	5.7 ± 6.2	<0.0001	8.6 ± 8.0	8.3 ± 7.5	0.0007	11.4 ± 9.4	11.1 ± 9.2	<0.0001	14.3 ± 11.0	13.9 ± 10.9	<0.0001

Patients monitored for lipid profile (%)	72.2	74.3	<0.0001	75.5	76.4	0.0002	74.9	75.4	0.07	67.3	69.0	<0.0001

Patients with LDL-C <100 mg/dL (%)	32.3	35.4	<0.0001	35.8	43.3	<0.0001	40.6	47.6	<0.0001	40.1	48.4	<0.0001

Patients with LDL-C ≥130 mg/dL (%)	34.9	31.7	<0.0001	31.6	25.4	<0.0001	27.0	20.7	<0.0001	26.8	19.6	<0.0001

Data are *n*, %, and means ± standard deviation.

**Table 3:** Management of LDL-C values in T2DM outpatients according to gender and diabetes duration.

	<2 years			2–5 years			6–10 years			>10 years		
	F	M	*P*	F	M	*P*	F	M	*P*	F	M	*P*
*n*	26736	36068		28475	37097		40272	52163		81651	89045	

Age (years)	64.4 ± 12.7	61.4 ± 12.1	<0.0001	65.5 ± 11.7	62.7 ± 11.4	<0.0001	67.2 ± 11.0	64.8 ± 10.5	<0.0001	71.3 ± 10.2	69.1 ± 9.8	<0.0001

Patients monitored for lipid profile (%)	69.8	71.4	<0.0001	74.7	76.4	<0.0001	74.4	75.9	<0.0001	72.4	74.3	<0.0001

Patients with LDL-C <100 mg/dL (%)	28.4	33.9	<0.0001	36.3	41.8	<0.0001	39.2	45.8	<0.0001	42.0	49.3	<0.0001

Patients with LDL-C ≥130 mg/dL (%)	41.6	34.9	<0.0001	30.9	25.6	<0.0001	27.6	22.1	<0.0001	24.9	19.2	<0.0001

Data are *n*, %, and means ± standard deviation.

**Table 4:** Age-adjusted and diabetes duration-adjusted indicators of quality of LDL-C management according to gender.

	F	M
Patients monitored for lipid profile (%)	73.2	74.4
LDL-C <100 mg/dL (%)	37.8	45.0
LDL-C ≥130 mg/dL (%)	29.2	22.9
Patients with LDL-C ≥130 mg/dL not treated with lipid-lowering medications	57.5	57.5
